# Wind-Direction Estimation from Single X-Band Marine Radar Image Improvement by Utilizing the DWT and Azimuth-Scale Expansion Method

**DOI:** 10.3390/e24060747

**Published:** 2022-05-24

**Authors:** Huanyu Yu, Hui Wang, Zhizhong Lu

**Affiliations:** 1 College of Intelligent Systems Science and Engineering, Harbin Engineering University, No. 145 Nantong Street, Harbin 150001, China; yuhuanyu@hrbeu.edu.cn; 2 Department of Electronic Information, Jiangsu University of Science and Technology, Zhenjiang 212003, China; wanghuijkd@just.edu.cn

**Keywords:** discrete wavelet transform (DWT), azimuth-scale expansion, occluded image, X-band marine radar, wind direction

## Abstract

In this study, a method based on the discrete wavelet transform (DWT) and azimuth-scale expansion is presented to retrieve the sea-surface wind direction from a single X-band marine radar image. The algorithm first distinguishes rain-free and rain-contaminated radar images based on the occlusion zero-pixel percentage and then discards the rain-contaminated images. The radar image whose occlusion areas have been removed is decomposed into different low-frequency sub-images by the 2D DWT, and the appropriate low-frequency sub-image is selected. Images collected with a standard marine HH-polarized X-band radar operating at grazing incidence display a single intensity peak in the upwind direction. To overcome the influence of the occlusion area, before determining the wind direction, the data near the ship bow are shifted to expand the azimuth scale of the data. Finally, a harmonic function is least-square-fitted to the range-averaged radar return of the low-frequency sub-image as a function of the antenna look azimuth to determine the wind direction. Different from the wind-direction retrieval algorithms previously presented, this method is more suitable for sailing ships, as it functions well even if the radar data are heavily blocked. The results show that compared with the single-curve fitting algorithm, the algorithm based on DWT and azimuth-scale expansion can improve the wind-direction results in sailing ships, showing a reduction of 7.84° in the root-mean-square error with respect to the reference.

## 1. Introduction

Ocean-surface wind information is a significant physical parameter in meteorology and oceanography, affecting most physical processes in the ocean. On ships, the measurement of wind parameters is of great significance for safe navigation and ocean engineering construction. Currently, in situ sensors located on the top of the ship are usually used to calculate this information, but the measurement of the wind parameters with the anemometer is affected by the superstructure. Even if the position of the anemometer is very good, the error of wind parameter measurements may be as high as 10% [1,2,3].

In recent decades, X-band marine radars have been widely used in sea-surface parameter retrieval [4]. The marine radar antenna emits X-band electromagnetic waves to the sea surface in a rotating manner. Due to the roughness of the sea surface, the electromagnetic waves are Bragg-scattered by sea-surface micro-scale waves [5], forming backscattering, and part of the backscattering is received by the radar antenna, which is called the sea clutter. Compared with conventional observations, marine radars can directly and continuously obtain sea-surface data and have stronger stability and safety. At present, X-band marine radars have been widely used to detect the current [6,7,8], bathymetry data [9,10,11] and waves [12,13,14,15], and significant progress has been achieved in this field. However, the extraction of wind parameters using X-band marine radars still needs further refinement.

The current ocean information retrieval technology has been comprehensively reviewed, and the feasibility of utilizing X-band marine radar to extract wind parameters and wave parameters was verified in [16]. Currently, there are two methods for retrieving the wind direction from X-band marine radar data. The first method uses the relationship between the radar return and the antenna look azimuth [17]. In the radar image, the azimuth of the wind direction forms a single peak, but the application of this method requires an unobstructed radar image of the entire 360° azimuths. The single-curve fitting algorithm developed in [18] uses a harmonic function that is least-square-fitted to the range-averaged backscattering intensity as a function of the antenna look direction. The upwind direction is given by the direction corresponding to the fitted function’s peak. Although the influence of the hull structure on radar occlusion is considered, the fitting is prone to large errors when the wind direction is near the ship bow azimuth in the radar image. In addition, the ocean is a random disturbance process with multiple models; the above method works well in the presence of wind waves but may not achieve good results in the presence of swell. The foam caused by breaking waves could have serious effects on the backscattering of electromagnetic waves and could affect the estimation results of the wind parameters. Subsequently, the method in [19] improved the method in [18] by adopting the dual-curve fitting, considering the blockage and island identification, and adding a curve fitting. Nevertheless, when the wind direction is blocked, additional curve fitting cannot work. The above method can retrieve wind direction well without rainfall, but in the case of rainfall, rain causes serious interference in the extraction of the sea-surface wind direction [20]. In [21], an ensemble empirical mode decomposition method (EEMD) was proposed to obtain the wind direction from the rainfall image with low wind speeds. However, this method still needs a complete 360° radar image and consumes a long calculation time. Subsequently, a new scheme for retrieving the wind direction was proposed from rain-contaminated X-band radar data in [22]. A self-organising mapping (SOM)-based clustering model is first used to identify rain-contaminated areas with fuzzy wave characteristics. Then, a convolutional neural network for image-haze removal is introduced for the rainfall correction of radar images. Finally, the mean azimuthal intensity of the rain-corrected radar images is curve-fitted to extract the sea-surface wind direction. The second method is based on the wind streak in radar images. In [23], the characteristics of wind streaks in the X-band marine radar were elaborated. They are low-frequency signals with a spatial scale of 200–500 m, and they are parallel to the wind direction. Based on the wind-streak theory, a motion estimation technique based on the optical flow method (OFM) [24] and the local gradient method (LGM) [25] were proposed. However, the OFM algorithm has a large error, and it is difficult to implement in engineering. The LGM requires the sub-sampling of wind-streak images, and accurate wind-direction results can only be obtained by reducing the image resolution to 1/16–1/4 of the wind-streak scale. On this basis, Ref. [26] put forward the energy spectrum method (ESM), which obtains the wave-number energy spectrum of small-scale wind streaks with a two-dimensional Fast Fourier transform. However, this method can only be used on fixed platforms. Even if multiple radar images are averaged in the case of ship-motion correction, there is a risk of obliterating the wind-streak signal. Moreover, this method needs to apply a three-dimensional Fourier transform to calculate the direction of the waves, which takes additional calculation time. Although the above methods can retrieve the wind direction from the X-band marine radar image, it is difficult to apply them in practical engineering due to the limitations of ship movement and fixed-object occlusion. Therefore, a technology that compensates for these shortcomings needs to be found.

The wavelet transform (WT) is now recognized as a useful and flexible technique for the ocean remote-sensing field in addition to the FFT-based method. The method in [27] uses the two-dimensional continuous wavelet transform (2D CWT) to analyze the spatial wavefield of the image. The comparison between the estimated values and the theoretical values of multiple wave parameters shows that the 2D CWT can identify the directional spectrum and wave characteristics in shallow water. Based on [27], the 2D CWT was used for ocean remote-sensing image analysis, and the ideal parameter value of the wavelet function was explored [28,29]. In [30], the 2D CWT was applied for wave measurement from X-band marine radar. Subsequently, in [31], the 2D CWT was used to detect the characteristics related to the marine atmospheric boundary layer and wind direction from SAR images. The discrete wavelet transform is the discrete form of the continuous wavelet transform, and it has the characteristics of the multi-resolution scale analysis [32]. In [33], the Haar wavelet transform (HWT) was used to decompose SAR images. The optimal threshold is used to describe the propagation direction of wind curls, and the wind direction is retrieved from these features. The results show that the discrete wavelet transform can also be applied to sea remote sensing.

In this study, the multi-resolution and multi-scale analysis of the DWT is applied to extract the low-frequency sub-image of the sea clutter image. In the low-frequency sub-image, interferences such as wave and noise are filtered out, and it has the characteristics of low-frequency static wind. Then, the sub-image with the appropriate scale is selected among the low-frequency sub-images of different scales. Compared with the method in [26], the wavelet transform of a single image can reduce the risk of wind features being obliterated due to ship movement. Finally, the wind direction is extracted from the relationship between the radar return and the antenna look azimuth. Before determining the wind, the data near the ship bow azimuth are shifted to expand the azimuth scale, which overcomes the shortcoming of data shortage caused by the shielding of fixed objects. This study is organized as follows: The data overview and data preprocessing are described in Section 2. The wind-direction retrieval algorithm is introduced in Section 3. The experimental results are illustrated in Section 4. Finally, a summary and future work are presented in Section 5.

## 2. Data Overview and Data Preprocessing

### 2.1. Data Overview

**Figure 1:**
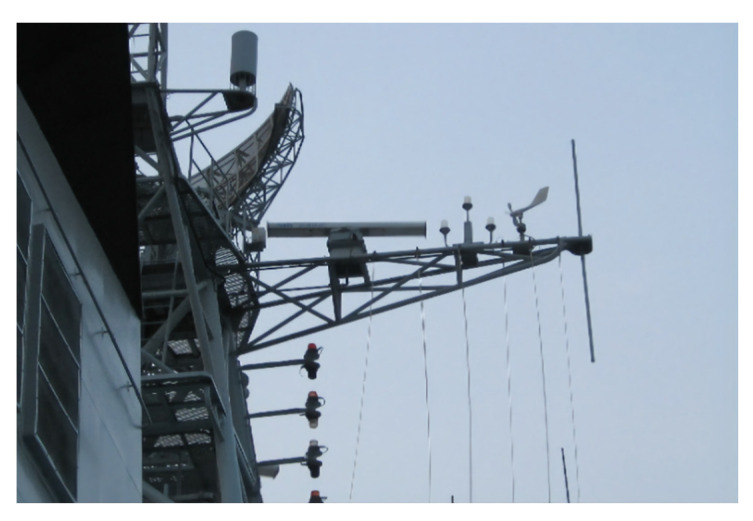
Radar installation position.

The data were collected from the Synapsis X-band marine radar, operating at grazing incidence with horizontal polarization (HH) in transmission and reception. The Synapsis marine radar works in short-pulse mode with a pulse width of 0.06 μs. As shown in Figure 1, the radar is installed on the ship’s mainmast, about 25 m above sea level, next to an anemometer for measuring wind direction and speed. The radar operates at 9.4 GHz with a sampling frequency of 20 MHz. The rotation period of the radar antenna is 2.5 s (24 r/min), and the pulse repetition rate is 3000 Hz. With this configuration, the radar image is collected every 2.5 s. The radar has a spatial resolution of 7.5 m in range and 1.3° in azimuth, covering an area with a radius of about ~4500 m. Each radar image sequence collected here consists of 32 images that represent a period of 80 s. The radar is connected to an HEU Wave Monitoring System, which digitizes the radar return information and stores it as a 14-bit grayscale depth image sequence with intensity ranging from 0 to 8192. In practical engineering, the gray value is converted to a 0–2.5 V voltage intensity value. For a point on the image, 0 corresponds to a voltage value of 0 V, and the radar return is the weakest; correspondingly, 8192 corresponds to a voltage value of 2.5 V, and the corresponding radar return is the strongest. The equipment synchronously collects original radar data as well as navigation information, such as heading and speed, and meteorological information.

In the actual data collection process, due to the interference of the external conditions (such as wind, ship roll) and the wear of its own components, the antenna speed was not very uniform, so the image data were not strictly collected every 2.5 s. In addition, due to some unpredictable situations during the test, the test was interrupted, which led to the discontinuity in some radar data. The reference wind data were used to verify the accuracy of the wind direction, which is measured by the shipborne marine radar. The main technical parameters of the anemometer are shown in Table 1. It measures and records the wind direction and speed every one minute.

**Figure 2:**
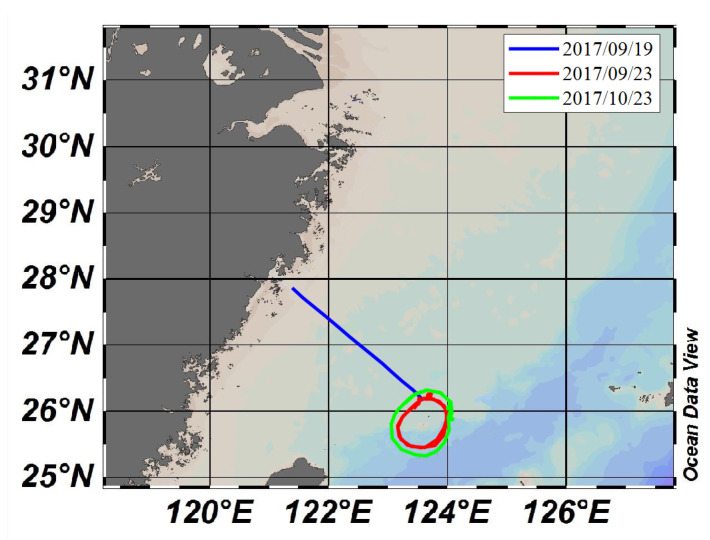
Ship’s course.

All the data used in this study are from sea trials conducted in 2017, mainly in the sea area between the coast of Zhejiang and Diaoyu Islands, and were provided by China Oceanic Administration. The corresponding ship’s course is shown in Figure 2. In the experiment, anemometer and marine radar data were collected during three periods from 19 September to 23 October 2017, for a total of 828 sets of data:

Period 1: from 19:15:22 19 September to 21:31:21 19 September 2017, with a total of 342 sets of data;

Period 2: from 00:01:00 23 September to 17:04:25 23 September 2017, with a total of 229 sets of data;

Period 3: from 16:04:16 23 October to 19:31:00 23 October 2017, with a total of 257 sets of data.

**Figure 3:**
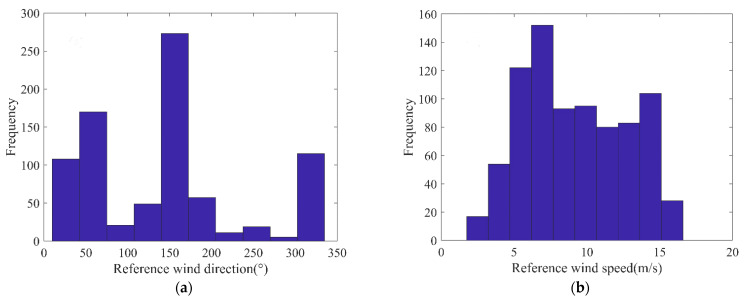
Distribution of reference wind direction and wind speed: (**a**) distribution of reference wind direction; (**b**) distribution of reference wind speed.

The data distribution of the reference wind direction and speed is shown in Figure 3. The wind direction ranged from 0° to 360°, including wind-direction data in almost all possible directions. The range of wind speed was 2~16.5 m/s, and the data between 6 and 8 m/s were the most abundant, but the data with wind speed less than 5 m/s were relatively few. In addition, in this study, radar data were randomly selected according to the wind direction; there were continuous radar image data. When the sea-surface wind wave changed rapidly, the time resolution of the anemometer was only 1 min, which may have led to errors in the final comparison results due to the sampling time difference.

### 2.2. Data Preprocessing

When retrieving the wind direction from radar images, co-channel interference, rainfall interference and solid object interference all reduce the imaging quality of a marine radar, thus reducing the inversion accuracy of wind direction. Before the discrete wavelet transform is performed on the X-band radar image, the data should be preprocessed to eliminate these interferences.

#### 2.2.1. Noise Processing

**Figure 4:**
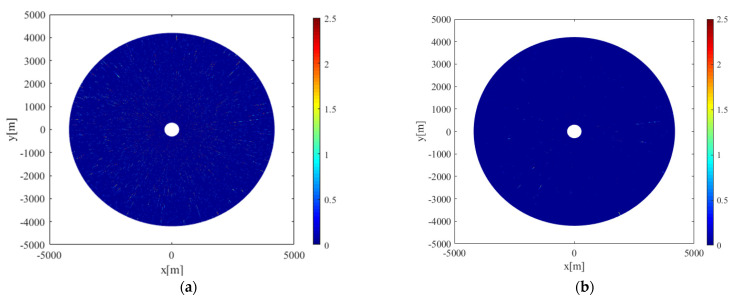
Radar image: (**a**) containing co-channel interference; (**b**) after filtering out the co-channel interference.

Figure 4a shows the original radar image of sea clutter containing co-channel interference. The striking lines of scattered distribution are the co-channel interference noise to be filtered. Co-channel interference changed the trend of the radar-return intensity, and its intensity value was significantly enhanced.

To remove the co-channel interference, a median filter was used to process the image. The median filter in this study selected a 3 × 3 moving template, and the radar image after the median filter is shown in Figure 4b. From Figure 4a,b it can be seen that we filtered out the influence of co-channel interference from the radar image and retained the characteristics of the radar image. For high-frequency noise, in the process of the discrete wavelet transform, high-frequency interference was filtered out by extracting its low-frequency components.

#### 2.2.2. Rain Recognition

**Figure 5:**
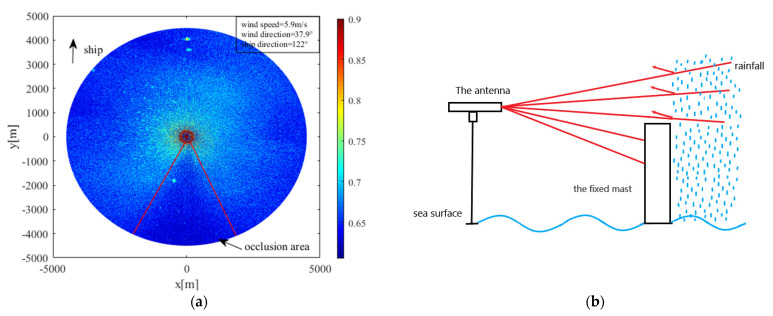
(**a**) Rain-contaminated radar image; (**b**) schematic diagram of the occlusion area.

Rainfall has a significant impact on the ocean surface, and the radar image associated with rain is seriously affected. As shown in Figure 5a, the radar return was enhanced by rainfall, and the features of sea-surface wind direction were partially blurred. Since the method used in this study is only suitable for rain-free radar images, the images had to be automatically distinguished according to the preprocessing method based on rain recognition. The radar used by the ship is blocked by a fixed mast, forming a fixed shadow area. In the X-band marine radar, when the propagation path of the radar electromagnetic wave is blocked by the mast, the radar antenna cannot receive the backward-scattered electromagnetic wave, resulting in low-intensity signals and fan shadow areas, which are occlusion areas in the image. When there is no rainfall, as it is difficult for the electromagnetic wave to spread to the sea surface behind the shelter, there is almost no radar return in the occlusion area. When there is rainfall, as shown in Figure 5b, raindrops in the sky can reflect the electromagnetic wave, resulting in rain echo in the occlusion area. As a result, the zero-pixel percentage (OZPP) of the occlusion area is determined as the quality control parameter to judge whether there is rainfall or not. The zero-pixel percentage formula, from [18], is

(1) $$P=\frac{f_{0}}{f}\;,$$

where f is the total number of pixels in the fixed occlusion area, and $f_{0}$ is the total number of zero-pixels in the fixed occlusion area. The data used in this study and those in [26] are from the same radar system; referring to its rainfall recognition threshold, 0.94 was selected as the threshold to detect whether the radar image was affected by rainfall. When the OZPP was greater than 0.94, it meant that there was no rainfall; when the OZPP was less than 0.94, the image was polluted by rain, and the contaminated image was discarded.

#### 2.2.3. Occlusion Area Processing

**Figure 6:**
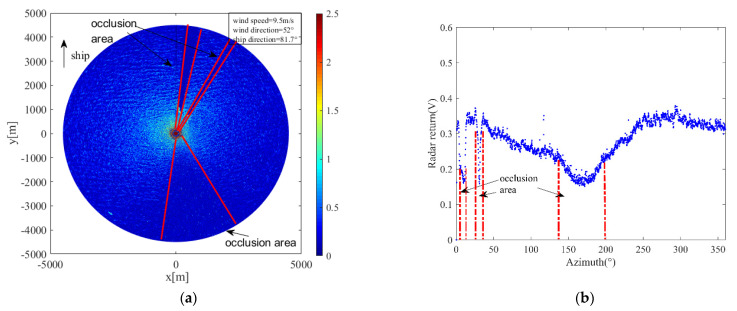
Schematic diagram of the occlusion area of the radar image: (**a**) occluded radar image; (**b**) corresponds to the radar-return intensity of (a).

For marine radar images, when the propagation path of radar electromagnetic waves is blocked by fixed obstacles such as islands and masts, the radar antenna cannot receive the electromagnetic waves, resulting in a large number of invalid signals and fan shadow areas, which are occlusion areas in the image. The existence of the occlusion area affects the distribution of the radar-return intensity. Before applying a wavelet transform to radar images, the range of the occlusion area should be excluded, otherwise it would affect the curve fitting and reduce the accuracy of wind-direction estimation. Figure 6a shows the radar image collected at 19:08 on 23 October 2017. The heading was 81.7°; the speed was about 12.5 knots; and the wind direction was 52°. The occlusion areas are marked by red lines in the figure. Figure 6b is the radar-return intensity of Figure 6a, which is expressed in the form of voltage. The abscissa represents the angle relative to the ship bow, and the ordinate represents the average radar-return intensity at the radar antenna look azimuth. It can be seen from Figure 6b that at the azimuth of about 138~195°, the radar-return intensity decreased sharply; an “abrupt change” occurred, and a small range of up and down fluctuations occurred. This is because the mast blocked the propagation path of the electromagnetic waves. At the azimuths of 4~12° and 29~34°, the value of the radar return was very low, which was caused by the occlusion of electromagnetic waves by islands during transmission. The radar return is greatly affected by the wind speed. Under different wind speed conditions, the threshold of the occlusion area is different. According to the observation of the radar-return intensity scatter diagram, to prevent the insufficiency of data caused by an excessive removal of occlusion areas, the appropriate threshold was selected to determine the occlusion area. In this study, when the wind speed was less than 10 m/s, the area with range-averaged radar-return intensity less than 0.25 V was identified as the occlusion area; when the wind speed was more than 10 m/s, the area with range-averaged radar-return intensity less than 0.35 V was identified as the occlusion area, and the data in the occlusion area were removed. When retrieving the wind direction, only the backscattering intensity points in the non-occluded area of the radar image were considered.

## 3. Wind-direction Retrieval Algorithm

### 3.1. Two-Dimensional DWT Description

In this study, wavelet analysis was implemented using the discrete wavelet transform (DWT). The DWT differs from the continuous wavelet transform (CWT) in that it limits the acceptability standard of the wavelet function to those that form orthogonal sets when scaling (stretching or compressing) with the power of 2, known as the binary scaling transform. Given a two-dimensional scaling function and a wavelet function,

(2) $$\varphi_{j_{0},m,n}\left(x,y\right)=2^{j/2}\varphi \left(2^{j}x-m,2^{j}y-n\right),$$

(3) $$\psi_{j,m,n}^{i}\left(x,y\right)=2^{j/2}\psi^{i}\left(2^{j}x-m,2^{j}y-n\right),\;\;i=\left\{H,V,D\right\},\;$$

where j,m,n∈Z, m and n determine the position of $\varphi \left(x\right)$ and $\psi \left(x\right)$ in the radar image, and j determines the width of $\varphi \left(x\right)$ and $\psi \left(x\right)$. The superscript i in Equation (3) indicates the wavelet in the horizontal (H), vertical (V) and diagonal (D) directions. Here, $\varphi \left(x\right)$ is a square-integrable function, denoted as $\varphi \left(x\right)\in L$^2^$\left(R\right)$. $\psi \left(x\right)$ is a class of functions with oscillatory characteristics that can rapidly decay to zero, i.e., $\int_{-\infty }^{+\infty }\psi (x)dx=0$. A function $\psi \left(x\right)$ that satisfies this condition is called a fundamental wavelet or mother wavelet. Then, the 2D DWT of a radar image $f\left(x,y\right)$ with a size M×N is defined as

(4) $$W_{\varphi }\left(j_{0},m,n\right)=\frac{1}{\sqrt{MN}}\sum_{x=0}^{M-1}\sum_{y=0}^{N-1}f\left(x,y\right)\varphi_{j_{0},m,n}\left(x,y\right),\;$$

(5) $$W_{\psi }^{i}\left(j,m,n\right)=\frac{1}{\sqrt{MN}}\sum_{x=0}^{M-1}\sum_{y=0}^{N-1}f\left(x,y\right)\psi_{j,m,n}^{i}\left(x,y\right),i=\left\{H,V,D\right\},$$

where $j_{0}$ is an arbitrary starting scale, and coefficient $W_{\varphi }\left(j_{0},m,n\right)$ is the approximation of $f\left(x,y\right)$ at scale $j_{0}$. Coefficient $W_{\psi }^{i}\left(j,m,n\right)$ details the horizontal, vertical and diagonal directions at scale $j\geq j_{0}$. The advantage of using standard orthogonal wavelet functions in the DWT is that the scales of the analysis do not overlap and can be computed efficiently using the Mallat algorithm [34].

**Figure 7:**
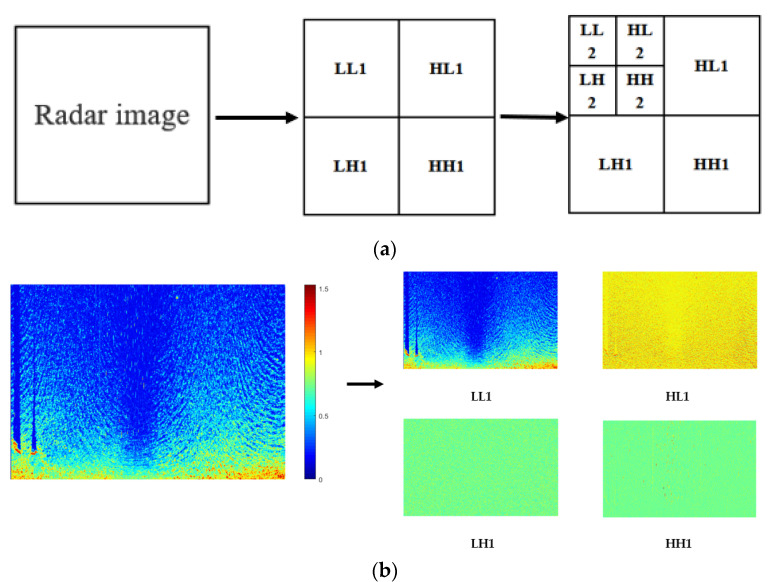
Two-dimensional wavelet decomposition: (**a**) first-level decomposition is shown on the left and second-level decomposition is shown on the right; (**b**) example of first-level wavelet decomposition of a radar image.

The radar image is a two-dimensional signal that can be filtered in the horizontal and vertical directions. In the 2D DWT process, two-channel sub-band coding is used. The 2D wavelet multi-resolution decomposition is achieved by two complementary filter banks (low-pass filter and high-pass filter) that generate wavelet coefficients using filter convolution in the horizontal and vertical directions, respectively. Where the low-pass filter gives an approximation of the signal and characteristics of the current low-frequency signal as well as obtaining the approximate contour characteristics, the high-pass filter gives the detailed values of the signal. As shown in Figure 7, the radar image is divided into four sub-components by first-level wavelet decomposition, which are the LL sub-component, corresponding to the low-frequency component of the image; the LH sub-component, corresponding to the lines’ low-pass- and then the columns’ high-pass-filtered versions of the image; the HL sub-component, corresponding to the lines’ high-pass- and then the columns’ low-pass-filtered versions of the image; and the HH sub-component, corresponding to the lines’ high-pass- and then the columns’ high-pass-filtered versions of the image [35]. The LL sub-component is further decomposed in the same manner in the second-level decomposition. Moreover, the 2D DWT is a multiresolution approach. When the DWT is applied to the image, it reduces the size of the image at every level to half of the previous level [36]. Therefore, an appropriate n-level decomposition must be determined.

### 3.2. Wind-Direction Extraction Process

**Figure 8:**
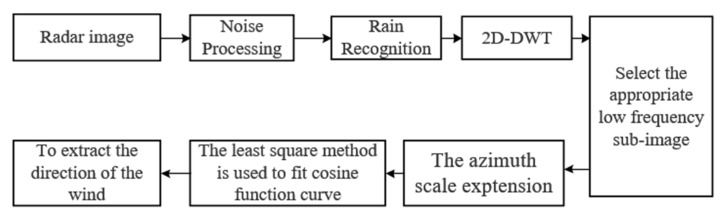
Wind-direction extraction process.

The process of wind-direction extraction is shown in Figure 8. To remove the co-channel interference, the image is processed by a median filter. The algorithm first distinguishes rain-free and rain-contaminated radar images based on the occlusion zero-pixel percentage and then discards the rain-contaminated images. According to the Mallat decomposition algorithm, the energy of the radar image can be completely represented by the energy of the expanded part in each frequency domain, namely, their expanded coefficients, so that the calculation of the wavelet transform is transformed into the calculation of the transformed coefficients [37]. In radar images, the sea-surface wind field with directional characteristics is a low-frequency signal relative to the waves, and it can be extracted by low-frequency component LL of the 2D DWT. This removes patterns that are highly variable in the spatial domain such as ocean-surface waves. In addition, the method for retrieving wind directions also considers the characteristics of wind-induced streaks in radar images. Most of these features are associated with wind streaks or streaks from foam or surfactants, which are in the scale from 200 to 500 m [26]. However, in marine-radar images, these wind streaks are superimposed by other ocean features and are barely visible. Since the wind streaks are parallel to the wind direction, the wind streaks in the upwind position in the radar image have some influence on the intensity of the radar return. When the wind streaks in the radar image happen to be in the wind direction, the radar return of this direction is more prominent than in other directions. To extract a more accurate sea-surface wind direction, it is necessary to make the image resolution and the scale of the wind streak reach a certain ratio. Since the scale of the small-scale wind streak is 200~500 m, it was testified in [25] that only when the image resolution is reduced to 1/16~1/4 of the wind-streak scale, the accurate direction of the small-scale wind streak can be calculated. The image resolution of the radar data used in this study was 7.5 m. After the three-level DWT (n = 3), the image resolution was reduced to 60 m, which was about 1/8~1/4 of the small-scale wind-streak scale. Hence, the three-level DWT was selected to extract the low-frequency components from the radar images. In this study, only the low-frequency coefficients of the radar image needed to be extracted. As shown in Equation (3), only the selected scaling function needs to be considered. The scaling function used is the Haar scaling function, and its definition is

(6) $$\varphi \left(x\right)=\left\{\begin{array}{c} \;\;1,\;\;\;\;\;0\leq x\leq 1\\ 0,\;\;\;\;\;\;\;\;\;\;\;\;\;\;\;else \end{array}.\;\;\right.$$

As one of the simplest scaling functions, the Haar scaling function can serve as a prototype for all other wavelets. In addition, it meets all the basic requirements of the Mallat multi-resolution analysis [32].

In this study, the data within the occlusion azimuth of fixed objects and other occlusion areas were excluded. For the X-band marine radar operating in the horizontal polarization, it is known that the backscattering intensity of the radar has only one peak in the upwind direction [16]. To retrieve the wind direction, it is necessary to average the radar-return intensity in the unobstructed antenna look azimuth; then, curve fitting is carried out according to the dependence on the radar return and antenna look azimuth. A harmonic function

(7) $$\sigma_{\theta }=a_{0}+a_{1}\cos^{2}\left(0.5\left(\theta -a_{2}\right)\right)$$

is least-square-fitted to the radar data [18]. Here, $\sigma_{\theta }$ is the range-averaged radar-return intensity as a function of antenna look azimuth θ, and $a_{0}$, $a_{1}$ and $a_{2}$ are the parameters determined by the curve fitting. The wind direction is determined by the antenna look azimuth corresponding to the peak point of the fitting function.

**Figure 9:**
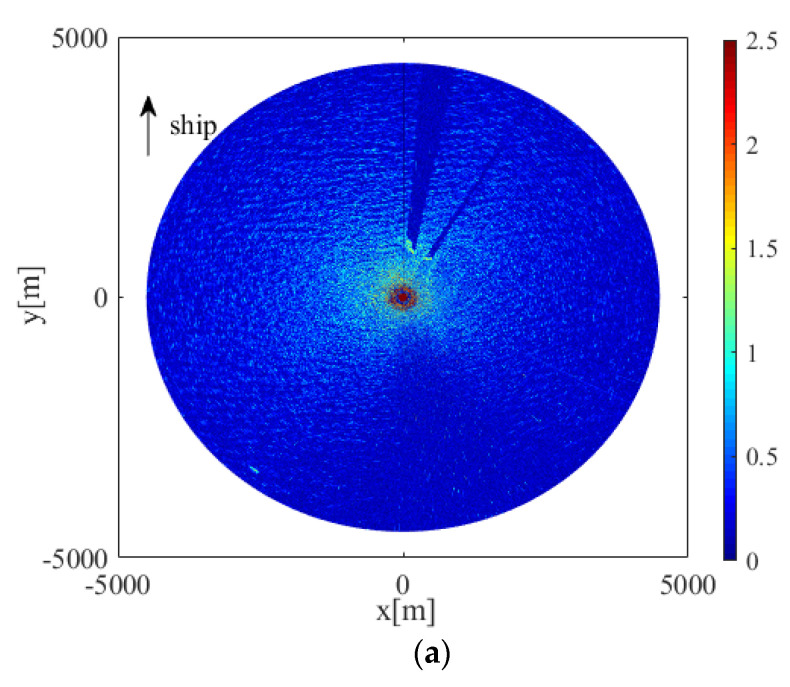

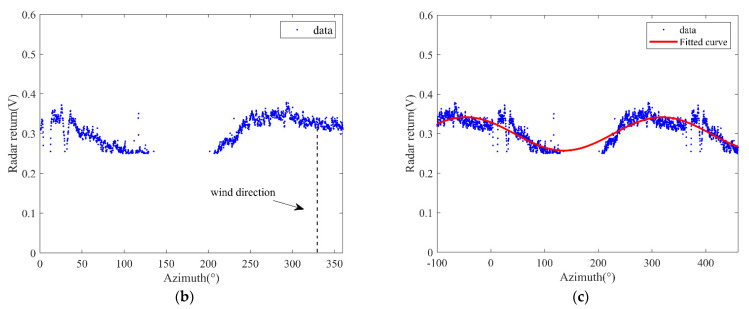
Example of the radar data (collected at 19:08, 23 October 2017): (**a**) backscattering image; (**b**) range-averaged radar return of non-occlusion region of (**a**); (**c**) range-averaged radar-return intensity as a function of antenna look direction after azimuth-scale extension.

The key to the retrieval of the wind direction is to find the peak point of the fitting function. However, the 0° azimuth in the scatter diagram is the ship bow; when the wind direction is near the ship bow azimuth in the radar image, as shown in Figure 9b, the data near the wind direction in the scatter diagram are separated to the azimuths of 0° and 360°. In the fitting process, even if the parameters are iteratively adjusted, it is difficult to find the peak point, and the error is large. Furthermore, the ocean is a multi-model stochastic perturbation process; the sea surface is very complex, and sometimes it is difficult to show ideal wind-field direction characteristics in radar images. As shown in Figure 9b, there is no obvious peak feature in the wind-direction position, and the range-averaged radar return near the wind-direction position is very large, which forms a large radar return of local azimuths. For this reason, an azimuth-scale extension method was used in this study that needs to use the data of the 100° azimuths. Due to the radar image data being distributed from 0° to 360° in polar coordinates, when the ship bow changes, the 0° azimuth of the starting point in the scatter diagram also changes. Therefore, when drawing the scatter diagram of the radar return, although the data of the 0° azimuth are separated, the data of the 0° azimuth and 360° azimuth are continuous in polar coordinates. Thus, the radar image data can be considered as continuous data in polar coordinates. After selecting a suitable radar sub-image, the data of the 0–100° azimuths were shifted to the 360–460° azimuths, and the data of the 260–360° azimuths were shifted to the −100–0° azimuths. Moreover, the data range of the fitting range of the function was extended to the −100–460° azimuths. The process of azimuth-scale extension is

(8) $$\sigma_{\theta }=\left\{\begin{array}{c} \sigma_{\theta +360{}^{\circ}\;\;},\;\;\;-100{}^{\circ}\leq \theta <0{}^{\circ}\\ \sigma_{\theta }\;\;\;\;\;\;\;\;\;\;,\;\;\;\;\;\;\;\;\;0{}^{\circ}\leq \theta <360{}^{\circ}\\ \sigma_{\theta -360{}^{\circ}\;\;\;},\;\;\;\;\;\;360\leq \theta \leq 460{}^{\circ} \end{array}\right..\;$$

As shown in Figure 9c, after the scale extension, the direction characteristics of the whole wind field are more obvious, and the changing trend of the radar return with the antenna look azimuth is clearer. In addition, the variation in the wind-related radar-return intensity with azimuth in radar images is a large-scale process, with radar return showing a single peak not only in the upwind direction but also rising and falling in other azimuths. The weighting of the beneficial orientation data is increased by azimuth-scale expansion after removing the effect of the occlusion area. This facilitates the fitting of the harmonic function to the sea-surface wind-field model, finding the peak point.

In the process of calculating the wind direction, the 0° azimuth begins with the ship bow azimuth, so the peak point obtained in the fitting is the azimuth of the wind direction relative to the ship bow, which is not the real wind direction. The relative wind direction needs to be converted to absolute wind direction, and the conversion formula is

(9) $$\theta_{wind}=\left\{\begin{array}{c} \theta +\theta_{ship},\;\;\;\;\;\;if\;\;\theta_{reference}\geq \theta_{ship}\\ \theta +\theta_{ship}-360{}^{\circ},\;\;\;\;\;\;\;else \end{array}\right.,$$

where $\theta_{wind}$ is the absolute wind direction; θ is the azimuth of the wind direction relative to the ship bow; $\theta_{ship}$ is the ship bow; and $\theta_{refrence}$ is the wind direction measured by the anemometer.

## 4. Experimental Results

**Figure 10:**
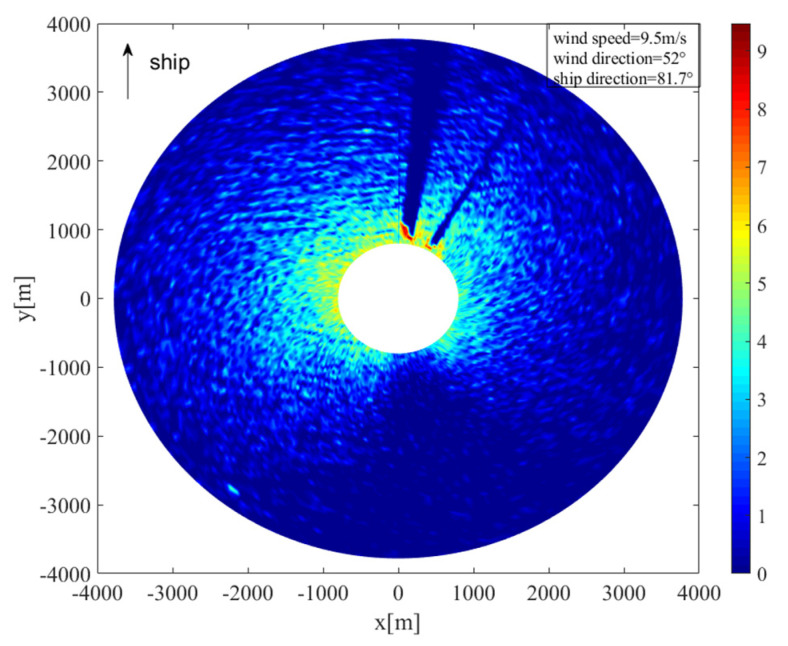
Third-level low-frequency component of the 2D DWT of Figure 9a.

The 2D DWT was performed on the radar image in Figure 9a, and the third-level low-frequency sub-image is shown in Figure 10. It could be found that the radar low-frequency sub-image extracted using the 2D-DWT did not have obvious wind-streak characteristics, whereas it retained the directional characteristics of the wind field in the radar image. The radar-return intensity had a strong dependence on the antenna look direction, which was the largest in the upwind direction, forming a local peak. The sea-surface wind direction was retrieved according to the selected low-frequency component. Before determining the wind direction, the data near the ship bow azimuth were shifted to expand the azimuth-scale range. Moreover, it was easier to find the upwind direction due to the increased weighting of data related to the wind.

**Figure 11:**
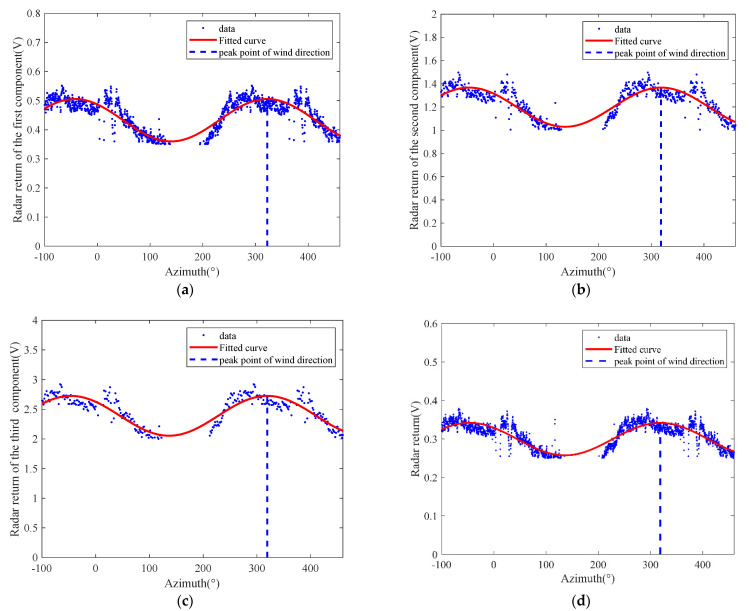
Curve fitting results: (**a**) two-dimensional DWT using the first low-frequency component and azimuth-scale extension-based method; (**b**) two-dimensional DWT using the second low-frequency component and azimuth-scale extension-based method; (**c**) two-dimensional DWT using the third low-frequency component and azimuth-scale extension-based method; (**d**) azimuth-scale extension method only.

**Figure 12:**
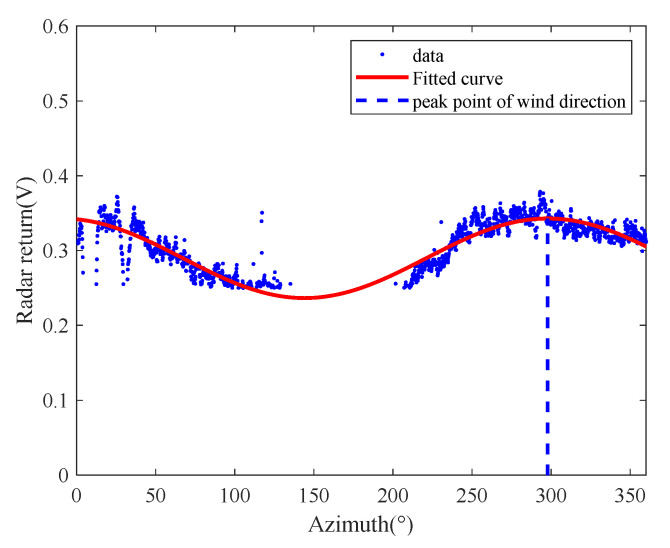
Single-curve fitting method.

The single-curve-fitting and the method based on the 2D-DWT and azimuth-scale extension described above were applied to the radar data after data pre-processing, and the results were compared to the reference data measured by a shipboard anemometer. The results of the method based on the low-frequency components from the 2D-DWT and azimuth-scale extension are shown in Figure 11a–c. The wind direction was determined to be 41.3°, 42.8° and 44.2°, with deviations of 10.7°, 9.2° and 7.8°, respectively, which were very close to the 52° measured by the anemometer. By comparing them with each other, the results obtained by the azimuth-scale extension method only is shown in Figure 11d, and the wind direction retrieved was 41°, with deviations of 11°. In addition, the result obtained by the single-curve fitting method is shown in Figure 12, and the wind direction retrieved was 20°, with deviations of 32°. The comparison shows that the azimuth-scale expansion method significantly improved the wind-retrieval accuracy by increasing the weight of the beneficial data. The single-curve fitting method produced large offset errors due to the shortage of data and the incompleteness of the sea-surface wind-field model. In particular, the effect was more pronounced when the wind direction was located near the ship bow direction in the radar image. The results of the wind statistics using the above algorithms are shown in Table 2. The method based on the azimuth-scale expansion only also improved the wind-direction results, showing a reduction of 5.89° in the root-mean-square error compared with the single-curve fitting method. The stability of the sea-surface wind-field model was improved due to the scale expansion. In addition, although the algorithm based on the 2D-DWT and azimuth-scale extension usually provides satisfactory wind-direction retrieval results, the third low-frequency component achieved the best result. The results from the second low-frequency component were worse than those of the first-level component. This was due to the DWT processing of the image, whereby each level of the image was reduced to half the size of the previous level. Therefore, the points of the second-level low-frequency component in the range and angle were fewer than those of the first-level low frequency component, which led to the divergence error of the scatter diagram. Moreover, after three-level DWT decomposition, the image resolution was reduced to 60 m, which is about 1/8~1/4 of the small-scale wind-streak scale. In this case, the radar return of the wind streak, which was hidden under the wave signal, enhanced the radar-return strength in the wind direction. Therefore, it achieved good results in the three-level DWT.

**Figure 13:**
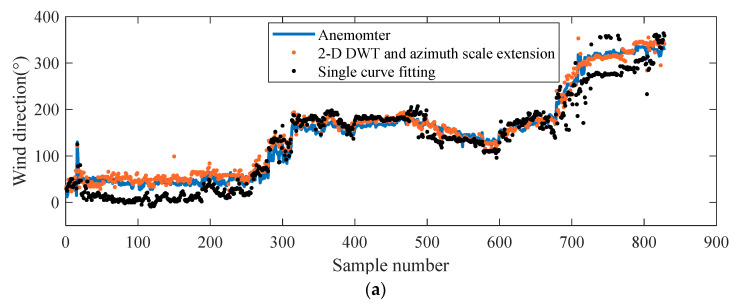

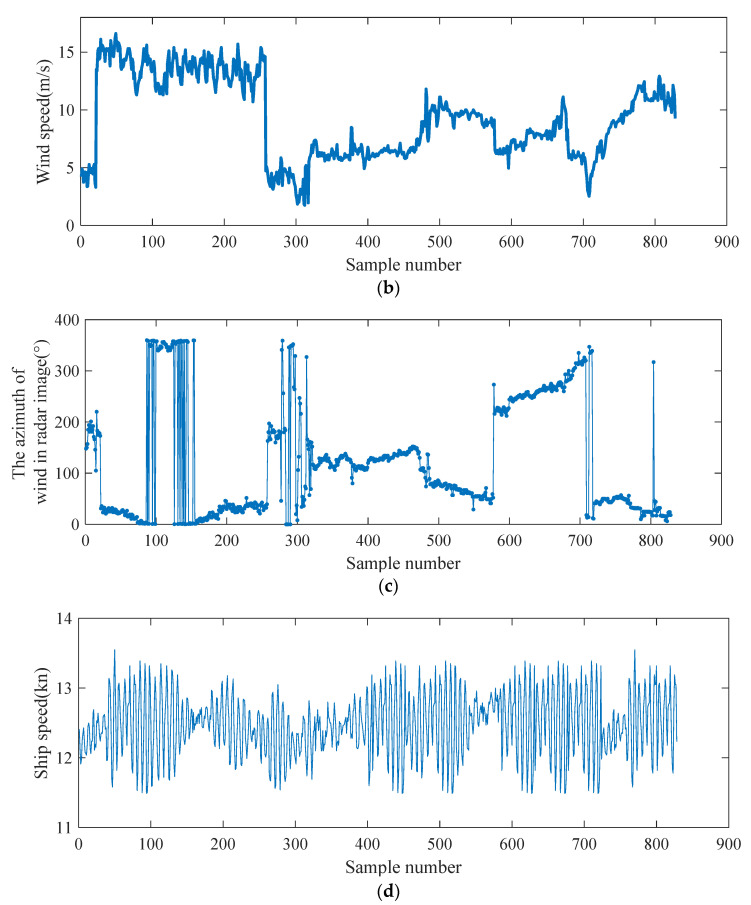
(**a**) Comparison of the two methods with the reference wind direction; (**b**) wind speed; (**c**) position of the wind direction in the radar image; (**d**) ship speed.

**Figure 14:**
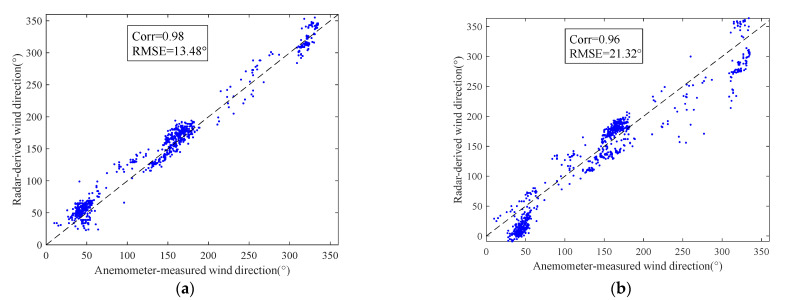
Scatter plots referring to Figure 13a: (**a**) method based on the 2D-DWT third-level low-frequency component and azimuth-scale expansion; (**b**) method based on the single-curve fitting.

Figure 13a shows the wind directions retrieved using the method based on the 2D-DWT with third-level low frequency and azimuth-scale extension and the wind directions retrieved using the single-curve fitting method, with all being compared with the reference data measured by the anemometer. It can be seen from Figure 13a that the results of these two methods were basically consistent with the anemometer. However, the method based on the 2D-DWT and azimuth-scale extension achieved better results. The wind speed value during the data collection period is shown in Figure 13b. The 2D-DWT-based method and the single-curve fitting method could retrieve the sea-surface wind direction very well, even in the case of low wind speed. Figure 13c shows the position of the reference wind direction in the radar image. In the fitting process, the position of the wind direction affects the curve-fitting accuracy. In some periods, the wind direction based on the single-curve fitting method had a large deviation from the reference data. As can be seen from Figure 13c, when the wind direction was distributed near the 0° azimuth of the radar image, it was easy to produce errors during fitting. On the contrary, the method based on the 2D-DWT and azimuth-scale extension could still provide satisfactory results. The shipping speed during this period is shown in Figure 13d. When the ship was sailing, stable results could be obtained, and the error between the wind direction and the reference data tended to be stable. This is because a radar image could be formed every 2.5 s, and the navigation of the ship had little effect on the imaging of a single radar. With reference to Figure 13a, the corresponding scatter diagrams of wind-direction inversion by the two methods are shown in Figure 14, respectively. It can also be seen that although the two methods could achieve satisfactory results, the method based on the 2D-DWT using the third-level low-frequency component and azimuth-scale extension was significantly better than the method based on single-curve fitting, with a correlation-coefficient increase of 0.02 and a root-mean-square error reduction of 7.84°.

## 5. Conclusions

In this study, a method based on the 2D DWT and azimuth-scale extension is proposed to retrieve the wind direction from a single X-band ocean radar image. The method is applied to radar images without rainfall. Firstly, the OZPP is used to detect rainfall and eliminate rainfall images. Then, according to the low-pass characteristics of the DWT and multi-scale analysis characteristics, the radar image with sea-surface wind-field characteristics is extracted from a single radar image using the 2D DWT. The three-level wavelet transform is selected according to the scale characteristics of the wind streak. The wind direction is given by a harmonic function which is least-square-fitted to the range-averaged radar return of the low-frequency sub-image. In the curve fitting process, the 100° azimuth data near the 0° azimuth are shifted to expand the scale of sea-surface wind field. The wind direction is determined by the antenna look azimuth corresponding to the peak point of the fitting function. The results show that compared with the method based on single-curve fitting, the method based on the azimuth-scale extension only could significantly improve the wind-direction retrieval results of occluded radar images, with the correlation coefficient being increased by 0.01 and the root-mean-square error being reduced by 6.09°. By azimuth-scale extension, the weight of the radar-return intensity associated with the wind direction is increased, and the scale of the whole wind field is consequently expanded, overcoming the fitting errors caused by the occlusion areas. Compared with the method based on azimuth-scale extension only, the method based on the 2D-DWT using the third low-frequency component and azimuth-scale extension could improve the wind-direction retrieval results of occluded radar images, with the correlation coefficient being increased by 0.01 and the root-mean-square error being reduced by 1.75°. Furthermore, the method only needs a single radar image, which consumes less calculation time, and can retrieve the wind direction in real time in sailing ships, so the method can be effectively applied in engineering.

Although the retrieval results of the wind direction are satisfactory, the 2D DWT-based algorithm still has many shortcomings. The radar return is greatly affected by the wind speed. Under different wind speed conditions, the threshold of the occlusion area is different. In the actual project, the rapid change in wind speed may cause excessive removal of the occlusion area, which may cause errors in function fitting. Nevertheless, the experiment in this study was carried out under the condition of relatively stable wind speed, so the occlusion area was easy to judge. Additionally, when the wind speed is high, it is often accompanied by rainfall. In the future, we also need to find an accurate threshold to determine the occlusion area. Meanwhile, retrieving the wind direction from the rainfall image is also a key issue to be studied in the future.

## Figures and Tables

**Table 1:** The main technical parameters of the anemometer.

Measured Parameters	Measuring Range	Accuracy of Measurement	Resolution
Wind speed	0~60 m/s	±0.3 m/s	0.1 m/s
Wind direction	0~360°	±3°	1°

**Table 2:** Wind-direction retrieval results.

Algorithm	DWT Low-Frequency Component	Correlation Coefficient	RMS Error (°)
Single-curve fitting		0.96	21.32
Azimuth-scale extension only		0.97	15.23
2D-DWT and azimuth-scale extension	1	0.97	14.12
	2	0.95	15.43
	3	0.98	13.48

## Data Availability

The raw/processed data required to reproduce these findings cannot be shared at this time as the data are also part of an ongoing study.
